# Era of Molecular Diagnostics Techniques before and after the COVID-19 Pandemic

**DOI:** 10.3390/cimb44100325

**Published:** 2022-10-11

**Authors:** Ahmad M. Alamri, Faris A. Alkhilaiwi, Najeeb Ullah Khan

**Affiliations:** 1 Department of Clinical Laboratory Sciences, College of Applied Medical Sciences, King Khalid University, Abha 61413, Saudi Arabia; 2 Cancer Research Unit, King Khalid University, Abha 61413, Saudi Arabia; 3 Department of Natural Products and Alternative Medicine, Faculty of Pharmacy, King Abdulaziz University, Jeddah 21589, Saudi Arabia; 4 Regenerative Medicine Unit, King Fahd Medical Research Center, King Abdulaziz University, Jeddah 21589, Saudi Arabia; 5 Institute of Biotechnology and Genetic Engineering (Health Division), The University of Agriculture, Peshawar 25130, Pakistan

**Keywords:** molecular diagnosis, COVID-19, PCR, clinical laboratories

## Abstract

Despite the growth of molecular diagnosis from the era of Hippocrates, the emergence of COVID-19 is still remarkable. The previously used molecular techniques were not rapid enough to screen a vast population at home, in offices, and in hospitals. Additionally, these techniques were only available in advanced clinical laboratories.The pandemic outbreak enhanced the urgency of researchers and research and development companies to invent more rapid, robust, and portable devices and instruments to screen a vast community in a cost-effective and short time. There has been noteworthy progress in molecular diagnosing tools before and after the pandemic. This review focuses on the advancements in molecular diagnostic techniques before and after the emergence of COVID-19 and how the pandemic accelerated the implantation of molecular diagnostic techniques in most clinical laboratories towardbecoming routine tests.

## 1. Molecular Diagnostics before Emergence of COVID-19

### 1.1. Timeline and History of Molecular Diagnosis—From Hippocrates to NGS

Molecular diagnosis deals with identifying the patterns and alterations in DNA and RNA. These genomic and proteomic patterns are used for comprehending and classifying the broad knowledge of prognosis, therapeutic monitoring, and diagnosis for human healthcare. Molecular diagnostic techniques are an amalgam of molecular techniques with human genetics and medical knowledge. It is a broad term comprising variable fields such as medicine, clinical pathology, forensic testing, epigenetics, immunotherapy, molecular oncology, metagenomics, molecular biology, biotechnology, immunosuppression, toxicology, precision medicine, etc. [1].

In the early 1980s, before the era of molecular diagnosis, clinical laboratories used human disease histories to cure health problems.The timelines of molecular diagnosis techniques are an incredible journey starting from the speculation of any transferrable material to next-generation sequencing technologies. The journey of molecular diagnosis has been reported back to the millennia of Hippocrates, who was credited as being the first person to speculate about the presence of any genetic material to be involved in the transfer. Fast-forward to 1866, the laws of Gregor Mendel demonstrated the inheritance patterns in pea plants [2]. The discovery of nuclei by Friedrich Miescher in pus cells and the isolation of the nucleus by Albrecht Kossel in 1878 started building the foundation for an upcoming era of research [3]. Meanwhile, the clinical approaches were also revolutionized in 1902 by the study of recessive patterns of disease inheritance discovered in alkaptonuria patients [4].

#### 1.1.1. The Invention of PCR

In the early 1990s, Oswald Avery’s transforming principle and Hershey–Chase experiments laid the foundation for comprehending the genetic transformation mechanism. The year 1953 is marked by a breakthrough in demonstrating the structure of DNA by Watson and Crick’s DNA model. With the isolation of DNA polymerase in 1958, the possibility of copying the genetic material was revealed. Karyotyping was first used to identify Down syndrome and trisomy 21, in 1959, and laid the foundation for analyzing the chromosomal aberrations[5]. The initiative of a significant wave of PCR invention in the history of science started with Khorana’s idea to synthesize a new gene using oligonucleotides.With the extraction of Taq Polymerase from Thermus Acquaticus, in 1976, molecular biology moved closer to achieving DNA replication [6].

The extraction of Taq Polymerase enabled Frederick Sanger to invent the Sanger sequencing method, in 1980, considered the first generation sequencing approach. Sanger sequencing works by incorporating random chain-terminating dideoxynucleotides during the process of DNA replication [7]. Finally, in 1985, Kary Mullis utilized the Sanger sequencing concepts in a productive way,which led to a significant wave of invention of PCR technology, which is a method of making billions of copies of desired DNA by using thermostable *Taq Polymerase*, extending the complimentary short sequences of primer under cyclic conditions [8,9].

#### 1.1.2. The Era of Next-Generation Sequencing

Following the advent of PCR, Applied Biosystems invented the first ground-breaking automatic sequencing machine AB370 with the capillary electrophoresis method to increase the proficiency of sequencing [10]. The next target was to crack the human genome for diagnosis and prognosis using the previously developed techniques. Myriad, the first molecular diagnostic company, formed in 1991, announced the development of BRCA analysis for detecting tumor suppressing genes, i.e., *BRCA1* and *BRCA2* in breast cancer.In the late 1990s, the development of kits to test hepatitis C, cytomegalovirus, BK virus, herpes simplex virus (HSV), and Epstein–Barr virus were innovated. The late 1990s have been marked by many advances in molecular diagnosis by clinical aspects such as amplificationand detection of nucleic acid simultaneously, the development of whole genome shotgun sequencing for targeting the bacterial influenza genome, and fluorescence in situ hybridization (FISH), which works on the principle of detecting the required sequence by hybridizing them with specifically designed fluorescent probes and, in 1999,detected the signal under thefluorescent microscope for detection of lungs cancer [11].

In the early 2000s, most works were already performed by providing the catalyst for an incoming explosive era of modern technology.The turn of the millennium saw massive advancements in information technology in the form of bioinformatics. Then came the era of ground-breaking and remarkable innovation of next-generation sequencing (NGS)with the arrival of NGS, the Genome Sequencer 20 (GS20), which was introduced in 2005–2007 by 454 Life Sciences corporation and became the first sequencer worldwide, later onworking on the process named 454 pyrosequencing [12]. Similarly, many sequencing technologies, such as ion semiconductor sequencing, were innovated in this era. This technique works on the principle of sequencing by synthesis. In this technique, the hydrogen ions releaseddue tothe polymerization of DNA are detected. When dNTPs are added in the microwell containing DNA template, they bind to complementary bases, and the hydrogen ions are released, which is detected by the ion sensor as an indication of reaction completion [13]. Single-molecule real-time sequencing (SMRT)was invented by Pacific Biosciences, in 2005. In this approach, each SMRT cell consists of thousands of zero-mode waveguides (ZMWs), providing the smallest detection volume in the world. When the ZMW is illuminated from below, attenuated light from the excitation beam enters the lower 20 nm of ZMW as the light wave is too large to pass through. This makes a powerful microscopic resolution having a detection volume of 20 zeptolitres. The DNA is immobilized in the SMRT chambers, and four differently labeled fluorescent nucleotides are introduced. The incorporation signal of nucleotides is detected by the signal detector [14]. Illumina/Solexa developed the sequencing by synthesis approach, in 2007 [15]. Similarly, another sequencing method known as combinatorial probe-anchor synthesis (cPAS) was developed and is a combinatorial approach to the sequence by hybridization and sequence by ligation. Sequence by hybridization, also known as ChiP-sequencing, consists of billions of oligonucleotides embedded on a surface to bind with their target complementary genomic sequences. In contrast, the sequence of ligation known as SOLiD sequencing uses the mismatch activity ofDNA ligases to detect the underlying nucleotide sequence of DNA [16]. *E.coli* genome was sequenced, in 2005, with 99.9% accuracy using Polony sequencing, which is a multiplex sequencing technique performed on paired-end tags library as a template for emulsion PCR on microbeads to produce the polonies or polymerase colonies [17]. Similarly, DNA nanoball sequencing was developed, which works on the principle of rolling circular replication (RCR) for producing amplicons of required genetic material into nanoballs. Helicos single-molecule sequencing, developed by Helicos Biosciences, works by adding poly-A tail adapters to the fragments of DNA molecules. The molecules are subjected to extension-based sequencing, andthe cycles wash away the fluorescent labels for the detection of signals [18].The year wise timeline of events and molecular techniques in diagnostics is represented in Table 1.

### 1.2. Some Molecular Diagnostic Tools Used in Clinical Laboratory before COVID-19

After the advent of NGS and Bioinformatics, molecular diagnostics in clinical labs started to grow. The simple and most common molecular diagnostic techniques used in the clinical laboratory were FISH, PCR, microarrays, MALDI-TOF, ELISA, and nucleotide sequencing [41].

FISH works on the principle of detecting the required sequence by hybridizing them with specifically designed fluorescent probes and detecting the signal under a fluorescent microscope. FISH is used to diagnose specific features in nucleic acids found in tumors, cancers, amniotic fluids, etc. PCR is used to rapidly amplify any target DNA or RNA of humans, virus, or bacteria, in determining pathogenic and non-pathogenic bacteria and in SNPs analysis. It is the basics of diagnostics in the clinical laboratory. Microarrays detect a large mass of genetic material with a high-throughput screening approach which involves multiplex assays mounted in silicon or glass substrates. The microarrays are used for a wide range of medical applications such as detecting chromosomal abnormalities, SNP detection, determination of post-translational modifications, copy number analysis, gene expression, mutation analysis, and finding causative agents of diseases. Many gene ChiP companies are developing microarrays, such as Illumina, Array IT, Agilent, Affymetrix,Applied Microarrays, etc [42]. Matrix-assisted laser desorption/ionization–time of flight, fully abbreviated as MALDI-TOF, is used to measure the amount of genetic material, and it works by crystallization of the PCR amplicon followed by ionization and detection of ions by the detector. It is widely used in genotyping, molecular typing, somatic mutation profiling, antibiotic susceptibility testing, differentiating Gram-positive and Gram-negative bacterial species, quantitative gene expression, methylation analysis, etc [43]. Another tool, enzyme-linked immunosorbent assay (ELISA) is used to detect hormones, peptides, proteins, and antibodies in blood serum by using antibody specificity and enzyme sensitivity. ELISA can also be used to detect the early stages of ovarian and breast cancer, HIV, New Castle Disease Virus (NDV), West Nile Virus, and hormone gonadotropin in pregnant women [41].

## 2. Advances in Molecular Diagnostic Tool after Emergence of COVID-19

With the emergence of COVID-19, the era of molecular diagnosis has undergone many improvements. Conventionally, CT scan, hematological assays, and RT-PCR were used. However, due to rapidly increasing cases of COVID-19 and the urgent requirement for rapid and precise testing, there was a dire need to come up with some inventions. For instance, CT scans cannot differentiate the type of virus and disease detection in asymptomatic patients. Additionally, this was costly and unavailable at all hospitals [44]. Similarly, RT-PCR was a widespread test that was used, but it was timeconsuming, expensive, and not sensitive enough to detect the low viral load of the virus in the early stages of infection [45]. Therefore, the researchers came up with novel approaches to detect coronavirus which were less timeconsuming and more costeffective.

### 2.1. Advances in Molecular Diagnostic Techniques in COVID-19

#### 2.1.1. Reverse Transcription Loop-Mediated Isothermal Amplification (RT-LAMP)

**Figure 1:**
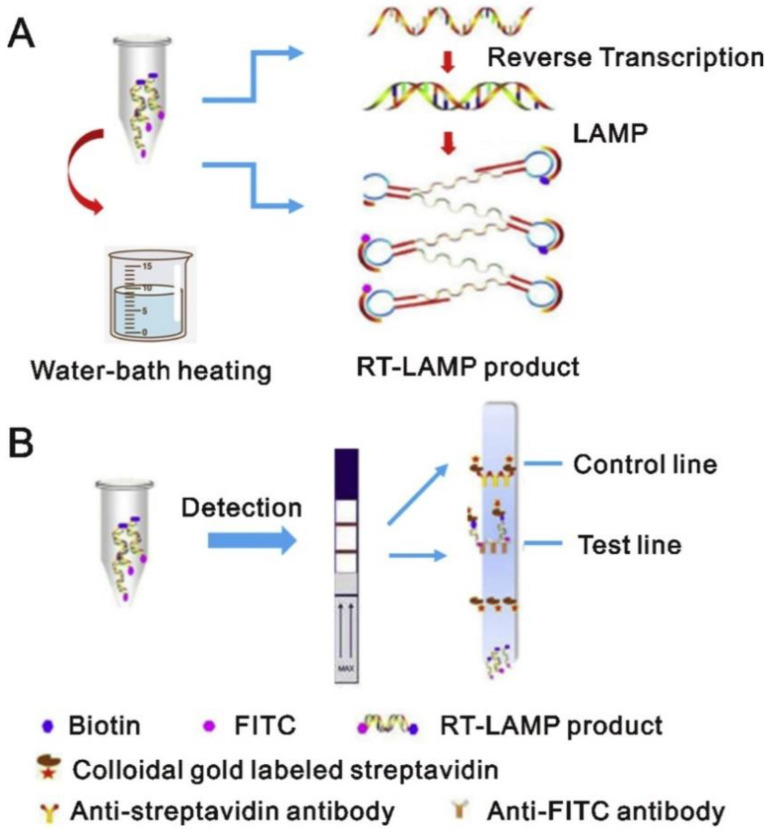
(**A**) Figure illustrates the working principle of RT-LAMP. RNA from the sample is extracted and converted to cDNA and then amplified using multiple cross-linked primers. Two loop primers, i.e., LF and LB, are labeled with fluorescein isothiocyanate and biotin, respectively. The amplicons, after replication, are labeled with biotin and FITC. A complex is made consisting of biotin-labeled amplicons with gold particles conjugated with streptavidin. While the anti-FITC antibody coated on the strip captures the complex conjugated with FITC. (**B**) The results can be observed visually on the test strip.

Loop-mediated isothermal amplification (LAMP) is an alternative to the cumbersome RT-PCR technique. It uses a single temperature, i.e., 60–65 °C, for amplification, which makes the technique much easier compared to RT-PCR and eliminates the need for a thermocycler, thus making it cheaper than RT-PCR. It can amplify the DNA in 25–35 min using polymerase with high DNA-strand replacement activity using 4–8 specific primers [46]. The high amount of DNA is amplified through this technique compared to conventional PCR. The LAMP reaction end products can be measured by either a fluorescent dye or by the turbidity of the sample, which relates directly to the viral content.Reverse transcription loop-mediated isothermal amplification, or RT-LAMP, uses reverse transcriptase to directly amplify RNA in a sample. Compared to RT-PCR and RT-LAMP, 76 nasopharyngeal samples demonstrated 97.6% sensitivity and 100% sensitivity, respectively [47]. According to recent reports, a novel single-tube real-time RT-LAMP assay has been developed with variable calorimetric versions with a limit of detection (LOD) of 119 copies per reaction [48]. Another robust modification of RT-LAMP has been developed, where samples from swabs can be directly used but with less sensitivity. Similarly, multiplex RT-LAMP combined with lateral flow biosensors has also been developed with high sensitivity and specificity [49]. LAMP can also be used for detection of SNPs for other diseases. Figure 1 illustrates the principle of amplification of SARS-CoV-2 and its detection [50].

#### 2.1.2. Biosensors

Biosensors are the advanced and robust, cost-effective, portable, and simple technology designed for the detection of various biomolecules such as pathogens, proteins, glucose, etc [51]. Several studies have reported the manipulation of this technology detection of SARS-CoV-2 [52,53]. Anti-microbial peptides are produced in response to foreign evading bacterial or viral peptides in organisms. For analysis, instead of using a whole protein, their small representative peptides can be used and coated with biosensors to increasing their stability and avoid the degradation of proteins.

##### Field-Effect Transistors (FET)

Many research studies have developed novel biosensors for detecting SARS-CoV-2, such as field-effect transistors (FET). FET is a biosensor developed for SARS-CoV-2, the surface of which is graphene coated. It is then conjugated with the SARS-CoV-2 anti-spike antibody through a probe linker. FET can detect approximately 1–100 fg/mL of the spike protein of SARS-CoV-2 with 2.42 × 10^2^ copies and LOD of 1.6 × 10^1^ pfu/mL [54]. Another spike protein (S1) of SARS-CoV-2 is detected with a novel biosensor developed on a bioelectric recognition assay. The LOD of this biosensor is 1 fg/mL, and the detection time is only 3 min. Additionally, this portable biosensor can be controlled via smartphone or tablet. Figure 1 illustrates the working principle of FET biosensors [55].

##### Localized Surface Plasmon Resonance (LSPR) Sensor

LSPR is a technique for producing optical phenomena when light waves are confined in gold nanoparticles. A coherent localized plasmon oscillation is produced when incident light and the surface electrons in the conduction band interact with each other [56]. Various viral sequences such as *E* genes, *ORF1ab* COVID, and *RdRp*-COVID were detected via application ofLSPR and a plasmonic biosensor using photothermal effect. In this process, the converted plasmonic photothermal energy is used to provide stable heat energy for increasing hybridization of *RdRp* of COVID-19 with the target complementary DNA sequence. The slope graph obtained from the LSPR with photothermal effect was observed to be higher than the LSPR without the photothermal effect, and sensors without PPT showed a false-positive outcome. This sensor has a LOD of 0.2 pM and can differentiate between S-CoV-2 and S-CoV with a high precision rate of 96% [57].

##### Cell-Based Potentiometric Biosensor

This technique detects the SARS-CoV-2 S1 antigen through the SARS-CoV-2 Spike S1 antibody. By using electro-insertion technique, a membrane-engineered kidney cell was modified with the S1 antigen of SARS-CoV-2. A change in potential when the antibody interacts with the required antigen is detected. This biosensor is formulated on eight gold screen-printed electrodes. These printed electrodes are shielded by a polydimethylsiloxane (PDMS) layer with approximately eight wells. A potentiometric device is used to measure the signal after the suspension of membrane and protein solution is added to the wells. The detection limit of this device is 1 fg/mL [52].

**Figure 2:**
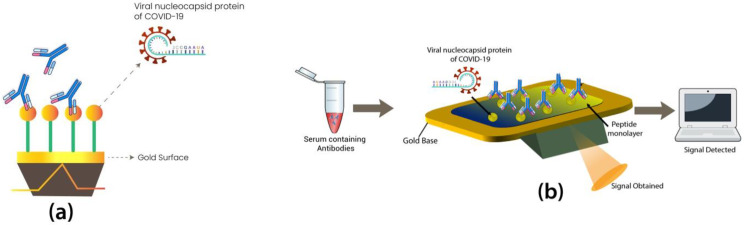
(**a**) Figure illustrates the working principle of SRP Biosensor. In this biosensor, the gold surface is coated with a single layer of viral nucleocapsid protein of SARS-CoV antigen to detect the anti-SARS-CoV-2 antibody present in the sample. (**b**) Figure illustrates the working principle of FET Biosensors, in which the isolated antibodies from the sample of patient is detected by SARS-CoV-2 antigens coated on the surface of sensor, and the signal obtained is detected by sensor.

Another single-step detection field-deployable biosensor using saliva samples was developed based on plasmonic fiberoptic absorbance with LOD of 10^−18^ M [58]. Similarly, another biosensor that works on the principle of immunoassay and aptamer-based technology (Figure 2) has also been established, named fiber optic surface plasmon resonance (FO-SPR) biosensor. An SPR sensor was made by coating the sensor with a monolayer of recombinant N Ag to detect anti-SARS-CoV-2 Ab, which gave results in 15 min [55].

#### 2.1.3. CRISPR-Based Diagnostics—SHERLOCK and DETECTR

Clustered regularly interspaced short palindromic repeats (CRISPR) is the most significant innovation of the era. It has induced many advances in molecular diagnostics. Previously famous for its use in gene editing, CRISPR has revolutionized the field of diagnostics after the COVID-19 outbreak. CRISPR-Cas is a part of the natural immune system of microbes for protection against foreign material by recognizing them and eliminating them via CRISPR-associated endonuclease Cas enzymes [59]. The two CRISPR-based innovative kits launched recently for SARS-CoV-2 detection are SHERLOCK and DETECTR.

##### SARS-CoV-2 DETECTR

In the SARS-CoV-2 DETECTR experiment, the extracted RNA from the test samples is subjected to RT-PCR to increasethe copy number of RNase P, E, and N genes. CRISPR-Cas12 detects the copies of genome sequences and illuminates with a fluorescence signal after cleavage of the reporter dye. This novel detection kit combines RT-LAMP, CRISPR-Cas, and Lateral Flow Assay (LFA) in one process. This CRISPR-based biosensor is developed by Mammoth Biosciences Inc. and Abbott Viral Diagnostics [60]. This technique showed 90% sensitivity and 100% specificity. The specificity of this technique is so high that it can detect the difference among SARS-CoV, SARS-CoV-2, and MERS-CoV differing with a single nucleotide sequence only due to highly specific primers and probes used. Similarly, AIOD-CRISPR and FELUDA are other modified versions of techniques using CRISPR in diagnosis [61,62].

##### Specific High Sensitivity Enzymatic Reporter UnLOCKing (SHERLOCK)

**Figure 3:**
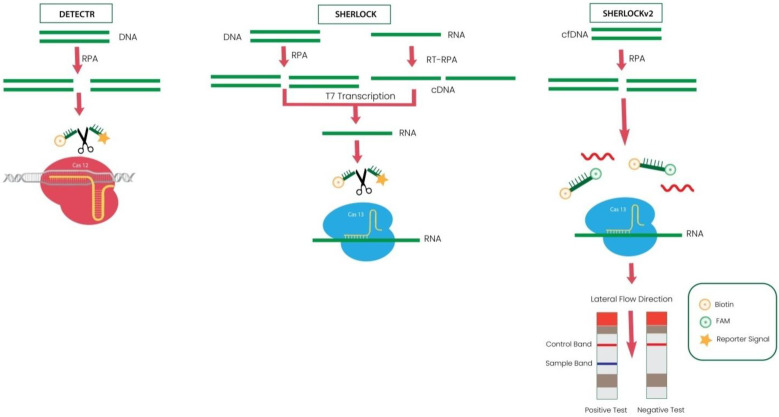
Figure illustrates the differences among DETECTR, SHERLOCK, and SHERLOCKv2. DETECTR (left) uses Cas12a for cleaving target DNA after RPA amplification. While SHERLOCK (middle) uses Cas13 programmed with crRNA to target ssRNA. DNA and RNA are amplified using RPA and RT-RPA, respectively. T7 transcription converts the DNA to RNA for processing by Cas13. SHERLOCKv2 (Right) uses cell-free DNA (cfDNA) and Cas13, Cas12a, Csm6. Direct visualization can be observed by lateral flow assay on strips.

The SHERLOCK system is based on the principle of the CRISPR-Cas VI system. SHERLOCK uses the Cas13 endonuclease activity from *Leptotrichiawadei* [63]. Recombinase polymerase amplification (RPA) is used to amplify target molecules Cas13 crRNA isothermally, and fluorescent RNA probes are mixed with amplified products [64]. If the amplified RNA products contain desired RNA, then Cas13 recognizes the desired RNA with the help of crRNA and cleaves the interaction between fluorophore and quencher. The cleavage of fluorescent signal results in illumination, and intensity of light directly depends on the quantity of the amplified sample. This technique is used to detect SNPs, Zika virus, pathogenic bacteria, and dengue virus [64]. Since the first SHERLOCK system was qualitative, the researchers developed SHERLOCKv2, a modified version. SHERLOCKv2 is 3.5-fold higher in sensitivity than the previous version, due to the combination of Cas13a with Csm6. The Csm6 endonuclease that supports the CRISPR type III can join Cas13a with the reporter signal, which enhances the signal quantity with diluted isothermal primers. Another advancement in SHERLOCKv2 is the development of commercial lateral flow strips to visualize colorimetric read-out. This version is fast, robust, sensitiveand superior to the previous one because it is a single-step assay in which unpurified samples can be directly applied to the strip without the need for purification and isolation [65]. Figure 3 illustrates the difference among DETECTR, SHERLOCK, and SHERLOCKv2.

#### 2.1.4. Aptamer-Based Diagnostics

**Figure 4:**
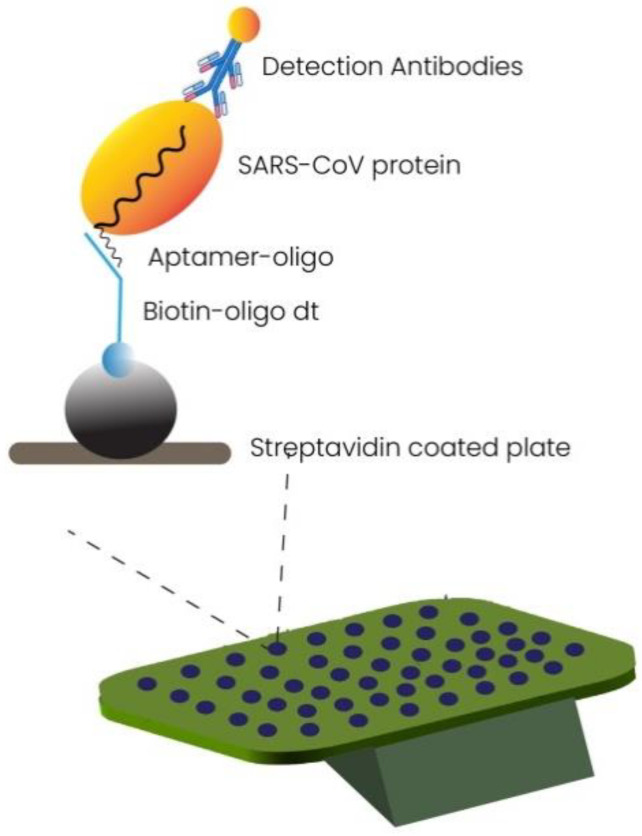
Figure illustrates the detection principle of SARS-CoV protein by aptamer-based technology. The streptavidin-coated plates,further coated with biotin labeled oligo (dT)_16_, are bound to aptamer oligo(A)_16_, which is attached to SARS-CoV N protein from patient sample. The detection antibodies such as polyclonal anti SARS-CoV N protein antibody bind to the N protein of SARS-CoV-2 for making complex and Alkaline phosphatase-conjugated goat anti-rabbit IgG antibody for measuring chemiluminescence.

Aptamers are artificially made small oligonucleotide or peptide sequences that target specific DNA or RNA of interest. Aptamer-based detection is an emerging technique to target viral infections [66,67]. Synthetic aptamers have been made to bind the SARS-CoV-2 receptor binding domain (RBD) specifically with high affinity using the human angiotensin-converting enzyme 2 (ACE2) competition-based approach. The small size of this biomolecule makes it a suitable candidate for a stable target in diagnostic techniques. The inhibitory potential of this small anti-RBD aptamer can be used in therapeutical treatments and diagnosis. Sensitive splint-based one-pot isothermal RNA detection (SENSR) is an RNA aptamer-based rapid detection approach. This technique works on the principle of ligation via SplintR ligase and T7 RNA Pol. The target RNA is amplified, and a fluorescent signal is detected. This single-step technique can detect a variety of pathogens with a detection limit of 0.1 aM [68,69]. Figure 4 illustrates the working of aptamer-based technology for detection of SARS-CoV-2. Figure 4 illustrates the working of aptamer based technology for detection of SARS CoV-2 [70].

#### 2.1.5. Molecular Imprinting Technology (MIT)-Based Diagnosis

**Figure 5:**
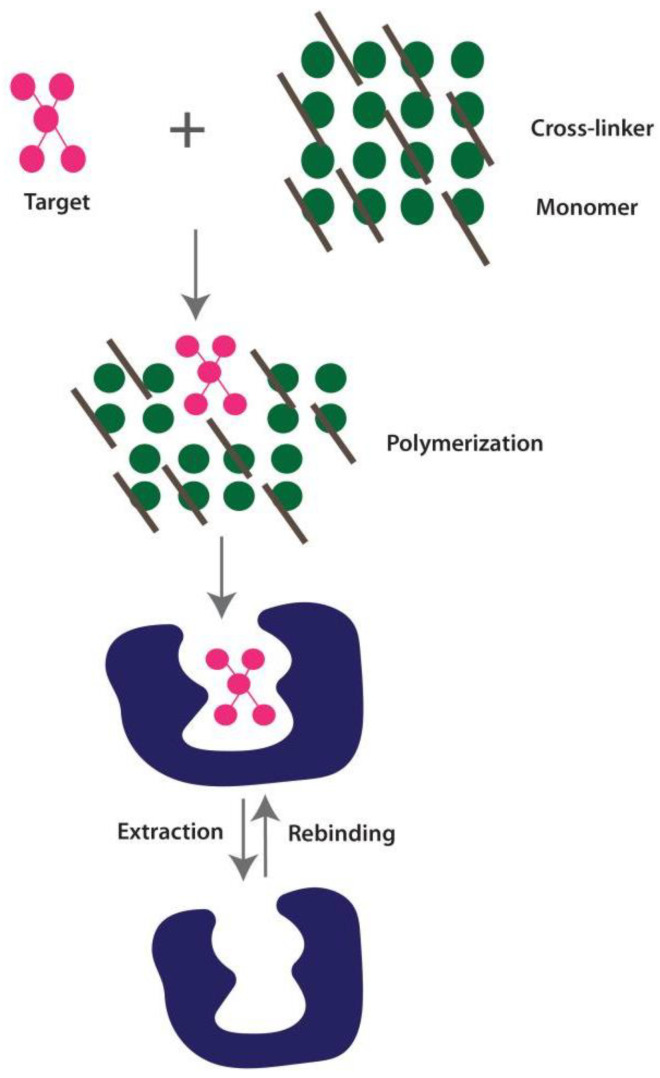
Figure illustrates the principle of molecular imprinting technology (MIT)- based diagnosis. A monomer and a cross-linker that surround the target molecule go through a process called polymerization to create MIP. This monomer–target molecule assembly is facilitated by both covalent and non-covalent interactions. The target molecule is complementary in shape to the MIP.

MIT is a diagnostic technique that works in the same way as the “Lock and Key Model” of enzyme and substrate reaction and binds to the most predictable structure with high specificity and affinity. MIPs are synthetically designed receptors for binding the complimentary target molecular of specific shape and orientation on a polymer [71]. The creation of MIPs with molecular recognition cavities that have specific selectivity for the template molecules is the basic principle. The formation of MIP involves the process of polumerization of a monomer and a cross-linker, both of which surround the target molecule. Covalent and non-covalent interactions promote this assembly of a monomer around a target molecule, as illustrated in Figure 5. A non-covalently produced MIP–target molecule complex is simpler to extract the target molecule from than a covalently formed MIP–target molecule complex [72].

MIPs have become widely employed in recent years for detection reasons, particularly for viral contamination. Ref. [73], recently reported the first work relating to the MIP-based detection of COVID-19. Utilizing a molecular imprinting approach, they created an electrochemical sensor for the detection of SARS-CoV-2 nucleoprotein. They showed that the nucleoprotein contained in nasopharyngeal swab samples from COVID-19-positive individuals could be detected by the MIP-based sensor. Their encouraging results support the need to create an effective, quick, and affordable MIP-based diagnostic tool for the identification of COVID-19 [73]. A rapid POC detection kit combined with SARS-CoV-2 specific aptamer and MIP sensor was proposed to detect the required target with greater affinity. Similarly, a MIP-based monoclonal antibody has been developed to bind SARS-CoV-2 selectively [74,75].

#### 2.1.6. Microarray-Based Diagnosis

**Figure 6:**
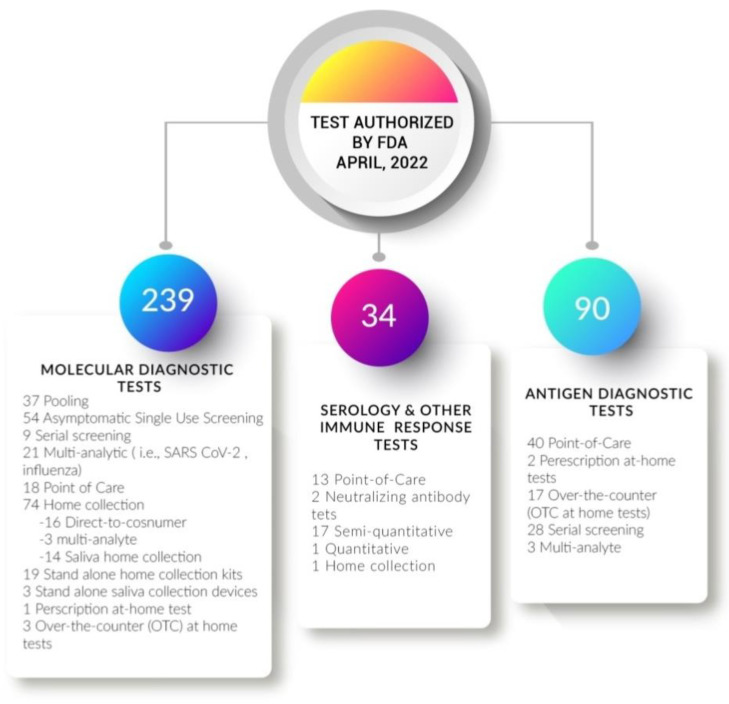
Figure illustrates the list of tests approved by FDA, in 2022. Approximately 239 new molecular diagnostic tests, 34 novel serology and immune response tests, and 90 antigen diagnostic tests have been approved by FDA.

Microarrays are multifunctional tools that are used in retrospective research on SARS-CoV-2. This tool can study antigen–antibody interactions, pathogenic behavior, cross reactivity between specific species and target proteins, and immunogenic responses to diseases [76,77,78]. In this high-throughput tool, the RNA of SARS-Cov-2 is converted into cDNA using RT enzymes, and the probes are conjugated with it. A microarray plate is then used to detect the hybridization of labeled cDNA with fixed oligonucleotides [79]. Researchers proposed that a SARS-CoV Ab response can be calculated using commercial antisera against SARS-CoV-2 proteins. A comparative study was performed among different respiratory viruses, and microarray tools can be used in determining antigen selection for diagnosis, vaccine development, and differences in variable pathogen-specific Abs (Figure 6) [80]. The advanced diagnostics of SARS-CoV-19 in development are represented in Table 2.

### 2.2. Development of New Kits for SARS-CoV-19 Detection

With the emergence of COVID-19 many advances in new techniques have been made as explained above. Similarly, many new kits have been approved by FDA for detection of SARS-CoV-19. The purpose of these kits is to provide rapid and robust testing with minimal time. In August 2021, a list of FDA approved many categories of kits varying on the principle such as RT-PCR kit, antigen detection kit, and antibody detection kit for detection of COVID-19 virus as shown in Table 3 [82].

In the history of molecular diagnosis, the SARS-CoV-2 outbreak significantly impacted the transformation and development of molecular diagnostic techniques. This pandemic enhanced the speed of research for survival. Many new technologies have been made, and more inventions are in the development pipeline. According to FDA reports of April 2022, 294 advancements in molecular tests, 90 in antigen depending tests, and 34 serological tests have been approved [83].

### 2.3. Point-of-Care Diagnostics

Point-of-care (POC) tests are the medical testing performed near the point of care where the point of care is the patient. POC are portable, cost-effective devices, with reduced sample processing eliminating the need to transport samples to the laboratory. POC does not require any trained professional to collect samples and can measure symptomatic and asymptomatic patients. Protecting the community from spreading any viral infection can be controlled only when detected early. POC offers the rapid detection of viral presence or host antibody response without laboratory settings needed.It can perform thousands of public tests in a single day. POC analysis can be performed at home, in offices, in mobile vans, in healthcare centers, and in emergency rooms [84]. For SARS-CoV-19, the RNA, antigen of the virus, and antibody produced in response to the virus by humans are detected using POC devices.

#### 2.3.1. Molecular Detection-Based Point-Of-Care Devices

The Isothermal amplification process is gaining much attention because it does not require the cycle at different temperatures and viral purification, thus making kits more rapid and easy to use. Many RT-PCR- and RT-LAMP-based POC devices have been developed since the emergence of COVID-19. Abbott Diagnostics Scarborough,Inc., developed the first RT-LAMP-based kit approved by EUA authorization, named the ID NOW COVID-19 test [83]. Similarly, another device, the Cue COVID-19 Test (EUA approved), works on the principle of isothermal amplification [85,86]. This device uses an electrochemical detection method and amplifies the RNA from direct nasal swabs in 20 min in a single step. Its LOD is 20 genomic copies per sample. Cue Health modified the kit for use at home on 5 March 2021. It became the first approved kit for use at home without prescription [87]. Cue COVID-19 Test (EUA approved) is another single-step rapid and robust battery-powered device that works on the RT-LAMP principle in 30 min using nasal swabs [88]. The list of some FDA-approved POC devices working on RT-PCR and RT-LAMP principles is shown in Table 4.

#### 2.3.2. Antigen Detection-Based Point-of-Care Devices

In antigen detection tests, specific antibodies against SARS-CoV-19 structural proteins are used. The antigen approach is faster and more rapid than PCR tests but has the drawback of being less sensitive because there is no amplification [89]. Thus, antigen-based tests are qualitative rather than quantitative because they cannot measure the viral load. It mainly targets the N-proteinsand S-proteins ofthe SARS-CoV-19 virus [90]. Ellume COVID-19 Home Test is the first antigen-based POC device authorized by EUA, in Dec 2020, for home use without the need for a prescription. Similarly, BinaxNOW COVID-19 Ag Card Home Test is another POC device approved for home use [91]. The list of some EUA-approved POC devices working on the antigen detection principle is shown in Table 5.

#### 2.3.3. Antibody Detection-Based Point-of Care Diagnostics

Antibodies produced bythe immune system in response to SARS-CoV-19 proteins can be detectedby using developed devices. They detect IgM and IgG antibodies from blood, serum, and plasma within 10–20 min. The list of some EUA-approved POC devices working on the antibody detection principle is shown in Table 6 [90].

## 3. Future of Molecular Techniques

Starting from the history to present, molecular diagnosis has revolutionized to a great extent with high-throughput technological advancements. It has become very convenient to study the entire genome with high accuracy and decreasing cost. Presently, many kits and tests are being developed which provide rapid detection as compared to the past, making molecular diagnosis indispensable in the clinical laboratory. As technology is overgrowing rapidly with time, the future of molecular diagnostics seems to be very promising. The wave of the corona accelerated and modernized the field of molecular diagnosis miraculously and analyzing the rapid growth in the pandemic assures us of a bright future ahead. The POC devices and biosensors can be potent candidates for molecular diagnosis because of their robustness. As the pandemics are likely to reoccur, investing efforts in molecular diagnosis seems highly imperative. As a result, the molecular diagnosis will be a central part of medical practice in the upcoming era. Whole genome sequencing will be the gold standard for identifying DNA variations associated with genetic diseases. Nevertheless, there is still a lot to discover before applying these techniques, such as cost-effectiveness, availability of resources, management of equipment, personnel training, and reproducibility. Along with proteomic-based testing, these developments will make further improvements in molecular diagnostics for implementation in hospitals, clinics, and healthcare departments, in public or private sectors.

## 4. Conclusions

The growth in molecular diagnostic labs before and after the emergence of coronavirus is notable. After the pandemic outbreak, the urge to develop fast and robust techniques exponentially increased. Many techniques have been modified to detect a massive community of coronavirus at home and offices. COVID-19 has boosted R&D struggles to launch innovative, more robust, rapid, precise and economical testing approaches than previously used tools. Currently, RT-LAMP, microarray-based detection, aptamer-based diagnosis, SHERLOCK, SHERLOCKv2, FET Biosensors, cell-based potentiometric diagnosis, molecular imprinting technology, etc. are used for diagnosis. Point-of-care (POC) diagnosis has rapidly increased to screen populations outside the hospitals. These POC devices are robust, portable, and can detect thousands of samples simultaneously. In previous times, the viral epidemic of SARS-CoV and MERS-CoV have led to the advancement in rapid diagnostics. For instance, the genome sequence identification of SARS-CoV-2 lead us toward developing nucleic acid-based diagnostic approaches, which assisted in controlling the outburst of COVID-19. The antibody-based and antigen-based diagnostic approaches helped in further comprehension. There is a lack of detailed research in the development of peptide- and aptamer-based biosensors, which could provide us more sensitivity and specificity in COVID-19 diagnosis, but the era of improvement has started, and yet, there is much to come. According to FDA reports, advancements in molecular tests, antigen-depending tests, and serological tests have been approved since the pandemic outbreak. Thus, the emergence has transformed the scientific community much more than any other life event. The scientific community’s collaboration to overcome the pandemic and break the chain of losing lives has increased the significance of molecular tools and molecular diagnosis.

## Figures and Tables

**Table 1:** Timeline of events and techniques in molecular diagnostics.

Year	Event/Invention	Reference
1949	Categorization of sickle cell anemia as a molecular disease	[19]
1957	Phosphonate synthesis assay for small oligodeoxynucleotides	[20]
1958	Isolation of DNA Polymerases by Arthur Kornberg	[21]
1960	Initial hybridization methods and electrochemical DNA Detection by Roy Britten	[20]
1965	Solid-phase oligodeoxynucleotide synthesis and Enzymatic synthesis of short RNAs	[20]
1969	Development of In situ hybridization technique by Gall and Pardue	[22]
1970	Isolation the first restriction enzyme and reverse transcriptase by Hamilton Smith	[23]
1970	Development of Nucleic acid hybridization methods	[24]
1975	Development of Southern blotting Technique	[20]
1977	Development of First Generation Sequencing technique-Sanger sequencing	[25]
1980	Maxim Gilbert Sequencing method	[26]
1985	Establishment of Restriction fragment length polymorphism analysis (RFLP)	[27]
1985	Invention of the polymerase chain reaction (PCR)	[9]
1985	Development of technique for detecting patient’s beta-globin gene for the diagnosis of sickle cell anemia	[28]
1986	Development of Fluorescence in situ hybridization (FISH)	[27]
1988	Discovery of the first thermostable DNA polymerase	[27]
1988–1991	Invention of first DNA Chip conceptions	[20]
1991	Designing of DNA/RNA mimics: peptide nucleic acid probes/PNA openers Ligase chain reaction; thermophilic DNA ligases	[20]
1992	Conception of real time PCR	[29]
1992	Assays for whole genome amplification and Strand-displacement amplification	[20]
1992	Development of Comparative genomic hybridization (CGH)	[27]
1993	Discovery of endonucleases for invasive cleavage assays	[27]
1994	Invention of DNA topological labeling	[20]
1995	Innovation of rolling amplification of circular probes	[20]
1996	First application of DNA microarrays	[27]
1996	Pyrosequencing technique-The next generation sequencing	[30]
1998	Lab-on-a-ChiP(microfluidics) for DNA analysis	[20]
1985–1999	Development of Immunoassays (Elisa, Western Blot, Immunostaining)	[20]
2000	Development of Massively parallel sequencing (MPS) by Lynx Therapeutics	[2]
2001	Application of protein profiling assays in diagnosis of human diseases	[27]
2002	HapMap project	[27]
2002	Development of Ion semiconductor sequencing	[31]
2005	Invention of Single molecule real time sequencing by Pacific Biosciences (SMRT)	[32]
2005	Invention of 454 Pyrosequencer system	[33]
2005	Invention of Polony sequencing by George M. Church	[34]
2005	Development of qRT-PCR, Virus microarrays	[35]
2006	Invention of Illumina/Solexa	[36]
2007	Invention of ABI/SoLID sequencing	[37,38]
2013	Invention of the CRISPR system	[39]
2014	Development of Portable oxford nanopore sequencing device	[40]
2015	Development of VirCapSeq-VERT	[35]

**Table 2:** SARS-CoV-19 advanced diagnostics in development [81].

Device Name	Platform	Sample Used	Developer	Status
CARMEN-Cas13a (Combinatorial Arrayed Reactions For Multiplexed Evaluation of Nucleic Acids)	- Microarray ChiP - 177840 micro wells - Supports more than 4500 tests	- Nasal swab - Throat Swab - Plasma - Amplified nucleic acid	Broad Institute, Harvard University	Considered for 169 human viruses
CRISPR-ChiP	gFET connected with a portable digital reader	- Buccal swab - Unamplified Nucleic Acids	Cardea Bio	- Detect disease associated mutations in less than 15 min - Specifically demonstrated for SARS-CoV-2 detection
CRISPR-Cas based Electrochemical microfluidic sensors	- Dry film photoresist layers piled on polyimide substrate - Contains an electrochemical cell which measures the H2O2 inversely produced to amount of target in sample	- Serum - Unamplified Nucleic Acids	University of Freiburg, Germany	- Developed for measuring microRNA in serum at low concentration - Specifically demonstrated for SARS-CoV-2 detection
Convat optical biosensor	A portable 25 × 15 × 25 cm box device controlled via tablet	- Antigen in POC test setup - Unamplified nucleic acid in multiplexed format - Nasal swabs - Saliva swabs	Catalan Institute of Nanoscience and Nanotechnology (Spain)	- Developed for detection of nosocomial bacterial pathogens - Currently used in SARS-CoV-2 detection
COVID-19 biosensor	Change in electrical resistance	- Viral antigen - Saliva	University of Utah	- Developed for detection of Zika Virus - Currently used in SARS-CoV-2 detection
Dual functional plasmonic photothermal biosensor (PPT)	Glass surface associated with gold nanoislands Functionalized with cDNA sequences	- Unamplified Nucleic acid - -Bioaerosol	Swiss Federal Institute of Technology in Zurich	- Developed for SARS-CoV-2 detection
FET Biosensors	gFET linked to a semiconductor analyzer	- Antigen with no pretreatment - Nasopharyngeal swabs	Korea Basic Science Institute	- Developed for SARS-CoV-2 detection
Femto Spot COVID-19 Rapid Diagnostic Test	Change in conductivity	- Antiviral IgG and IgM - One drop of untreated blood	Nano DiagnosiX	- Developed for detection of cardiac biomarkers - Currently used in SARS-CoV-2 detection
One-step COVID-19 test	- Fluorescent read out ≤1 h	- Viral nucleic acid - Nasal swab - Saliva swab - Environmental samples	Northwestern University, Stemloop	- Developed for detection of environmental pollutants - Currently used in SARS-CoV-2 detection
VIRRION (virus capture with rapid Raman spectroscopy detection and identification)	ChiP consisting of N-doped C nanotube arrays with gold nanoparticles for increasing Raman spectroscopic signals	- Whole virus - Nasopharyngeal swabs - Exhaled breath	Pennsylvania State University	- Developed for detection of influenza A virus subtypes and humans respiratory infections - Currently used in SARS-CoV-2 detection

**Table 3:** List of kits for SARS-CoV-19 approved by FDA 2021.

	Kit Name	Developer	Sensitivity	Specificity
List of Permitted PCR Based Test Kits for Commercial Use				
1	SARS-CoV-2 Fluorescent PCR Kit	Maccura Biotechnology Co., Ltd.	96.23%	100%
2	BioFire Respiratory Panel 2.1 (RP2.1)	BioFire Diagnostic, LCC.	100%	100%
3	DENSY PACK UNIVERSAL REAGENT (i-DENSY PACK UNIVERSAL SARS-CoV-2 DETECTION SYSTEM)	ARKRAY INDUSTRY INC.	100%	100%
4	SARS-CoV-2 DETECTION PRIMER PROBE SET REAGENT (i-DENSY PACK UNIVERSAL SARS-CoV-2 DETECTION SYSTEM)	ARKRAY INDUSTRY INC.	100%	100%
5	SARS-COV-2 Nucleic Acid Detection Kit (PCR-Fluorescent Probe Method)	Zybio Inc.	100%	100%
6	Xpert Xpress SARS-CoV-2	Macare Medicals, Inc	100%	99%
List of Permitted Antigen Test Kits for Commercial Use				
1	PanbioTM COVID-19 Ag Rapid Test Device	Abbot Rapid Diagnostics Jena GmbH	CT < 30 (97.83%)	100%
2	PanbioTM COVID-19 Ag Rapid Test Device	Abbot Diagnostics Korea Inc	CT < 30 (97.83%)	100%
3	SOFIA 2 SARS Antigen FIA	Quidel Corporation	CT < 30 (92.86%)	100%
4	PanbioTM COVID-19 Ag Rapid Test Device	Abbott Rapid Diagnostics	CT < 30 (97.83%)	100%
5	STANDARD™ Q COVID-19 Ag TEST KIT	SD Biosensor, Inc	CT < 30 93.1%	100%
6	PanbioTM COVID-19 Ag Rapid Test Device	Abbott Rapid Diagnostics	CT < 30 (90.5%)	100%
7	NowCheck COVID-19 Antigen Test	BioNote Inc-22	CT < 30 (91.4%)	100%
8	Novel Coronavirus (2019-nCoV) Antigen Detection Kit (Colloidal Gold Method)//Wondfo2019-nCoV Antigen Test (Lateral flow)	Guangzhou Wondfo Biotech Co.,	CT < 30 (92.2%)	100%
List of Permitted Antibody Rapid Test Kits for Commercial Use				
1	NADAL COVID-19 IgG/IgM Test	Nal Von Minden GmbH- Carl-Zeiss-Str. 12, 47,445 Moers, Germany	92.67%	100%
2	VivaDiagTM COVID-19 IgM/IgG Rapid Test	Vivachek Biotech Hangzhou Co. Ltd.	92.00%	99.33%

**Table 4:** List of EUA-approved POC devices working on RT-PCR/RT-LAMP for SARS-CoV-19 detection.

Kit Name	Principle	Approved
Xpert Xpress SARS-CoV-2	RT-PCR	EUA-approved
Xpert Xpress SARS-CoV-2/Flu/RSV	RT-PCR	EUA-approved
Xpert Xpress SARS-CoV-2 DoD	RT-PCR	EUA-approved
Accula SARS-CoV-2 Test (Mesa Biotech Inc.)	RT-PCR	EUA-approved
Cobas SARS-CoV-2 and Influenza A/B Nucleic Acid Test (Roche Molecular Systems, Inc.)	RT-PCR	EUA-approved
BioFire Respiratory Panel 2.1-EZ (BioFire Diagnostics, LLC)	RT-PCR	EUA-approved
Visby Medical COVID-19 Point-of-Care Test (Visby Medical, Inc.).	RT-PCR	EUA-approved
Visby Medical test	RT-PCR	EUA-approved
ID NOW COVID-19 test	RT-LAMP	EUA-approved
Cue COVID-19 Test	RT-LAMP	EUA-approved

**Table 5:** List of EUA-approved POC devices working on antigen detection principle for SARS-CoV-19 detection.

Kit Name	Principle	Approved
LumiraDx SARS-CoV-2 Ag Test(LumiraDx UK Ltd.)	Antigen detection	EUA-approved
CareStart COVID-19 Antigen test (Access Bio, Inc.)	Antigen detection	EUA-approved
BinaxNOW COVID-19 Ag Card (Abbott Diagnostics Scarborough,Inc.),	Antigen detection	EUA-approved
BD Veritor System for Rapid Detection of SARS-CoV-2 (Becton, Dickinson and Company, LLC)	Antigen detection	EUA-approved
BD Veritor System for Rapid Detection of SARS-CoV-2 (Becton, Dickinson and Company, LLC),	Antigen detection	EUA-approved
QuickVue SARS Antigen Test	Antigen detection	EUA-approved
Sofia 2 SARS Antigen FIA	Antigen detection	EUA-approved
Sofia 2 Flu + SARS Antigen FIA (all three from Quidel Corporation)	Antigen detection	EUA-approved
Status COVID-19/Flu (Princeton BioMeditech Corp.)	Antigen detection	EUA-approved
Ellume COVID-19 Home Test	Antigen detection	EUA-approved
BinaxNOW COVID-19 Ag Card Home Test	Antigen detection	EUA-approved
QuickVue At-Home OTC COVID-19 Test	Antigen detection	EUA-approved

**Table 6:** List of EUA-approved POC devices working on antibody detection principle for SARS-CoV-19 detection.

Kit Name	Principle	Approved
Assure COVID-19 IgG/IgM Rapid Test Device (Assure Tech.)	Antibody Detection	EUA-approved
RightSign COVID-19 IgG/IgM Rapid Test Cassette (Hangzhou Biotest Biotech)	Antibody Detection	EUA-approved
RapCov Rapid COVID-19 Test (Advaite, Inc.)	Antibody Detection	EUA-approved
MidaSpot COVID-19 Antibody Combo Detection Kit (Nirmidas Biotech, Inc.)	Antibody Detection	EUA-approved
Sienna-Clarity COVIBLOCK COVID-19 IgG/IgM Rapid Test Cassette (SalofaOy)	Antibody Detection	EUA-approved

## Data Availability

Not applicable.

## Abbreviations

NGS	Next Generation Sequencing
PCR	Polymerase Chain Reaction
HSV	Herpes simplex virus
FISH	Fluorescence In Situ Hybridization
ZMWs	Zero-mode wave guides
SMRT	Single-molecule real-time sequencing
cPAS	Combinatorial Probe-Anchor Synthesis
RCR	Rolling Circular Replication
ELISA	Enzyme-linked Immunosorbent Assay
NDV	New Castle Disease Virus
RT-LAMP	Reverse Transcription Loop-Mediated Isothermal Amplification
LAMP	Loop-Mediated Isothermal Amplification
LOD	limit of detection
FET	Field-Effect Transistors
LSPR	Localized Surface Plasmon resonance
PDMS	polydimethylsiloxane
FO-SPR	Fiber Optic Surface Plasmon Resonance
CRISPR	Clustered Regularly Interspaced Short Palindromic Repeats
RPA	Recombinase Polymerase Amplification
RBD	Receptor binding domain
SENSOR	sensitive splint-based one-pot isothermal RNA detection
ACE2	Angiotensin-converting enzyme 2
MIT	Molecular imprinting technology
POC	Point-of-care
